# Pseudomembranous Cystitis: An Uncommon Ultrasound Appearance of Cystitis in Cats and Dogs

**DOI:** 10.3390/vetsci8070125

**Published:** 2021-07-02

**Authors:** Caterina Puccinelli, Ilaria Lippi, Tina Pelligra, Tommaso Mannucci, Francesca Perondi, Mirko Mattolini, Simonetta Citi

**Affiliations:** Department of Veterinary Sciences, University of Pisa, Via Livornese Lato Monte, 56121 Pisa, Italy; caterina.puccinelli@phd.unipi.it (C.P.); ilaria.lippi@unipi.it (I.L.); pelligratina@gmail.com (T.P.); tommy.mannucci@gmail.com (T.M.); f.perondi87@gmail.com (F.P.); mirko.mattolini@phd.unipi.it (M.M.)

**Keywords:** pseudomembranous cystitis, cat, dog

## Abstract

In veterinary medicine, pseudomembranous cystitis (PC) is a rare condition described only in cats. The purposes of this retrospective study were to describe ultrasound features of PC in cats and dogs, predisposing factors, comorbidities and outcomes. Cats and dogs with an ultrasonographic diagnosis of PC were included in the study. The bladder ultrasound findings that were recorded were: pseudomembranes’ characteristics, abnormalities of the bladder’s wall and content and anomalies of the pericystic peritoneal space. Ten cats and four dogs met the inclusion criteria. Four pseudomembrane adhesion patterns were described. The presence of pseudomembrane acoustic shadowing was observed in the 60% of cats. A total of 80% of the cats included were presented for urethral obstruction (UO) and/or had at least one episode of UO in the previous 2 months. Thirteen patients out of fourteen received only medical therapy, and all of them survived. PC is a rare disorder in cats and dogs and there are some ultrasonographic differences between the two species, suggesting a greater severity of the pathology in cats. Chronic cystitis and UO may have a potential role in the development of feline PC. Finally, the medical approach can be a non-invasive and effective approach for PC.

## 1. Introduction

In veterinary medicine, pseudomembranous cystitis (PC) is a rare condition described only in cats [1]. This pathology is associated with severe diffuse ulceration, necrosis, and hemorrhage of the bladder wall, with intraluminal necrotic, fibrinous and hemorrhagic material, as already described by Le Boedec et al, in four cats with urine outflow obstruction [1]. At the ultrasound examination, the urinary bladder presents a peculiar ultrasound feature, represented by multiple hyperechoic luminal septa and/or strips resembling membranes, associated to urinary bladder wall thickening and intraluminal suspended echogenic debris [1]. Similar ultrasonographic findings have also been found in one case report and one study about cats with lower urinary tract obstruction [2,3]. Finally, the ultrasound appearance of PC was also observed in dogs [4], but at the moment, to the authors’ knowledge, there are only one study and one book describing PC in this species [5]. In human medicine, the definition of PC is controversial. Indeed, it is a term used to describe a specific form of chronic cystitis, similar to that described in cats, where pseudomembranes, composed of shaggy layers of necrotic, gray or yellow cell detritus, fibrin, inflammatory cells and blood, cover a hemorrhagic and ulcerated mucosa [6], but also to describe non-malignant inflammatory metaplastic changes in the bladder trigonal epithelium, particularly observed in females, more commonly defined as pseudomembranous trigonitis [7,8,9].

The etiology and pathogenesis of PC are still not well understood. In cats, a possible correlation between PC and urethral obstruction (UO) and bacterial infection was hypothesized [1,3]. In humans, various origins of bladder wall necrosis have been described, including bacterial infections [10,11,12,13], drugs such as cyclophosphamide [6,14] and ischemia of the bladder wall due to excessive and prolonged bladder distension [10,11,15,16,17]. For pseudomembranous trigonitis, several explanations have been proposed, including, first of all, a hormonal impact related to the presence of estrogen receptors in the trigone [8,18,19,20]. 

Regarding the therapy, a surgical and a medical approach were described in cats, which were both successful. The first one was described by Le Boedec et al., and it consisted of a cystotomy with the removal of the intravesical material, associated with a post-operative medical treatment [1]. On the other hand, the medical approach was described by Vila et al., which prevalently included fluid therapy with lactated Ringer’s solution, amoxicillin–clavulanic acid, buprenorphine, prazosin and urethral catheterization [3].

The aims of the present study were to describe ultrasound features of PC in both cats and dogs, and to evaluate possible predisposing factors, comorbidities and outcomes.

## 2. Materials and Methods

The medical records of client-owned cats and dogs of various breeds, genders, and ages, referred to the Veterinary Teaching Hospital of the Department of Veterinary Science (University of Pisa, Pisa, Italy) between January 2015 and December 2020, with an ultrasonographic diagnosis of PC, were retrospectively reviewed. For each patient, data regarding signalment, history, including the presence of previous episodes of UO, physical examination, clinical signs, urinalysis findings (bacteriuria, leukocyturia, hematuria and crystalluria), urine culture when available, therapy and outcome were collected from medical records. We also registered how many other cats and dogs had an ultrasonographic aspect suggestive of cystitis in the same period.

Ultrasonography was performed using a Canon Aplio a CUS-AA000 (Canon Medical Systems Europe B.V., Zoetermeer, the Netherlands), with a 7.5 MHz microconvex probe and a 12 MHz linear probe. Ultrasonographic records, images and videos were reviewed for each patient by a single radiologist (S.C.). 

An ultrasound diagnosis of PC was based on the presence of intraluminal hyperechoic septa and/or strips resembling pseudomembranes. The pseudomembranes were classified into four ultrasonographic patterns, based on their different type of adhesion to the bladder wall, as described in Table 1.

The eventual presence of associated acoustic shadow was also evaluated. Moreover, ultrasonographic abnormalities of the bladder wall and content and anomalies of the pericystic space were recorded.

Finally, the presence of kidney and/or ureteral ultrasonographic anomalies was also registered.

For each patient, the type of received therapy was recorded, and classified as medical or surgical.

The outcome was evaluated by reviewing the medical records and/or by phone interviews with the owners. Patients that presented at least one episode of clinical signs related to cystitis within six months after the diagnosis of PC were considered to have a recurrence of cystitis.

Statistical analyses were performed using commercial statistical software (GraphPad Prism 5.0, GraphPad Software Inc, San Diego, CA, USA). Normality for quantitative variables was assessed by the Shapiro–Wilk test. Descriptive statistics were calculated, and the median and range were reported for each variable.

## 3. Results

A total of 14 patients were included (10 cats and 4 dogs) and the ultrasonographic diagnosis of PC in our study, over a 5-year period, corresponded to the 0.03% (10/335) of the ultrasonographic diagnosis of cystitis in cats, and to the 0.004% (4/895) of the ultrasonographic diagnosis of cystitis in dogs. 

All cats were mixed breed and male; nine cats were castrated, and one cat was intact. The median age was 3.5 years (range, 2–12 years). All the dogs were pure breed (one Bichon Frisé, one Cavalier King Charles, one Jack Russell Terrier and one Pug); there were two castrated females, one castrated male and one intact male. The median age was 6 years (range 1–15 years). 

**Figure 1:**
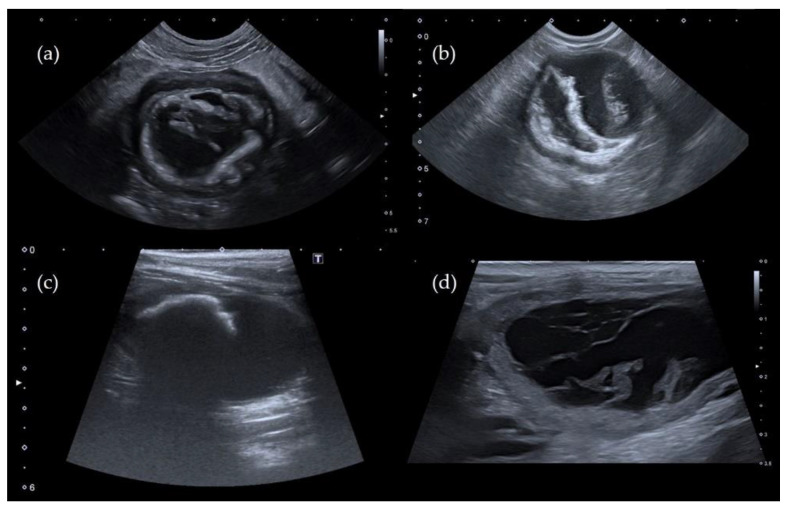
Ultrasound images of the urinary bladder showing the different types of adhesion pattern in three cats (image **a**–**c**) and one dog (image **d**). (**a**) Type 1; (**b**) Type 2; (**c**) Type 3; (**d**) Type 4.

Different adhesion patterns of the pseudomembranes were present both in cats and dogs (Figure 1), and an association with acoustic shadowing was observed in 60% of cats, as shown in Table 2. In dogs, the “complete adhesion pattern” and the association with acoustic shadowing were not detected in any patient.

Diffuse bladder wall thickening and intraluminal echogenic sediment were present in all the cats and dogs (Table 2).

**Figure 2:**
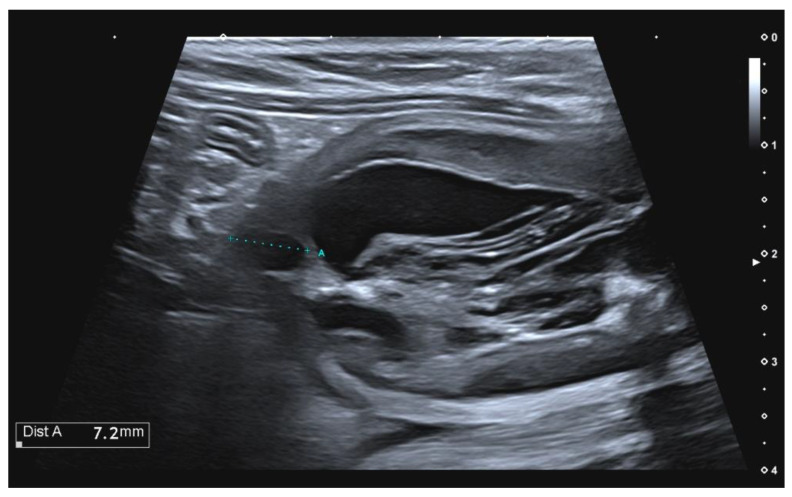
Longitudinal ultrasound image of the urinary bladder in a cat, showing the presence of a small, fluid-filled, anechoic structure, in the cranial bladder wall, showed by the cursor indicated with the letter “A”, consistent with intramural vesicourachal diverticulum.

A vesicourachal diverticulum was observed in three out of ten cats (Table 2). In two subjects, the vesicourachal diverticula were intramural (Figure 2), and in one cat, the vesicourachal diverticulum was extramural.

In cats, the presence of hyperechoic pericystic fat was the most frequent ultrasound abnormality for the pericystic peritoneal space; instead, hyperechoic pericystic fat and pericystic effusion were observed in only one dog (Table 2).

Concerning the kidneys and the ureters, the most common finding in cats was pyelectasia. This finding was observed in 60% of cats, of which four out of six patients were presented for UO and one out of six patients had concomitant ureteral dilatation associated with nephrolithiasis and ureterolithiasis. Hyperechoic perirenal fat and retroperitoneal effusion were observed, respectively, in 50% and 30% of cats, where retroperitoneal effusion was always associated with the presence of hyperechoic perirenal fat. Regarding the canine group, in only one patient were pyelectasia and ureteral dilatation observed.

All the cats were hospitalized. The reasons for hospitalization in cats were UO in five out of ten patients and intermittent UO due to continuous straining in the remaining patients. Three of these last patients had at least one episode of UO in the previous two months. 

For the dogs, three of them were referred for the presence of symptoms related to cystitis and one of them for polytrauma. Two out of four dogs were hospitalized, but only one as a consequence of the urinary disease. 

All the cats presented both non-specific general clinical signs (depression and anorexia or poor appetite) and specific urological signs (hematuria, pollakiuria and stranguria). Instead, only one dog had a concomitant presence of general and specific clinical signs (anorexia, stranguria and pollakiuria), and the remaining three dogs only presented hematuria.

All patients had urinalysis available for review, which confirmed the presence of hematuria in all cases.

Crystalluria and leukocyturia were present in five out of ten cats, while bacteriuria was present in two out of ten cats. In dogs crystalluria, leukocyturia and bacteriuria were always present. The urine culture was available for five out of ten cats and one out of four dogs; the exam resulted positive only in one cat (Staphylococcus sp >100 CFU/ml). 

According to the management, four out of four dogs and nine out of ten cats received medical treatment. For hospitalized patients, medical management included pain medication (buprenorphine or methadone iv), iv fluids (type and rate of fluids were set according to individual hydration status), antibiotics (amoxicillin–clavulanate 12 to 15 mg/kg by mouth three times a day or enrofloxacin 5 mg/kg iv once a day), and a closed system urinary catheter in cats with UO. Antibiotic treatment was performed in seven out of ten cats and one out of four dogs. Amoxicillin–clavulanate was the molecule of choice in five out of seven cats, while in two out of seven cats, enrofloxacin was continued, as previously introduced by the referring veterinarian. For the canine group, only one of the two hospitalized dogs received amoxicillin–clavulanate, due to the presence of more severe clinical signs. 

Urinary catheters were kept in situ for the minimum time possible (3 days maximum), to maintain the patency of the urethra. When hematuria was present, bladder irrigation was performed twice to three times a day, to promote the removal of blood clots and intravesical debris. The decision to remove the urinary catheter was based on the macroscopic appearance of urine. The catheter was removed as soon as macroscopic hematuria reduced significantly, and/or intravesical debris was no longer detected in the closed urinary system. During hospitalization, all patients were fed with their usual diet (both dry and wet). No diet change was performed during hospitalization, to avoid any food aversion. Only one out of the ten cats received an association of medical and surgical therapy, which included the medical approach described previously, followed by cystotomy with the removal of the intravesical material. In this patient, the histological examination of the urinary bladder wall was performed. The histological diagnosis was suggestive of ulcerative cystitis, with the presence of ulceration of the urothelium involving the submucosa, associated with phenomena of necrosis and fibrin deposits. All patients hospitalized were discharged from the hospital. The median time of hospitalization in cats was 4 days (range 3–8 days). The dog hospitalized for symptoms related to cystitis was discharged after 5 days. The dog presented for polytrauma had a longer hospitalization (13 days) due to the management of pelvic fractures. At hospital discharge, owners were asked to carefully monitor the patients for any signs of UO and to progressively increase the water content of diet. When possible, a urinary-specific diet was slowly introduced. 

A follow up was available for all patients. For the cats, six out of ten patients recovered completely, which included the patient who received surgical therapy; four out of ten had recurrence of cystitis, of which one of them had a new episode of UO at 13 days post-discharge. For the dogs, two out of four patients recovered completely and two out of four had a recurrence of cystitis.

An ultrasonographic examination at follow up was available in one cat and two dogs within a period between 15 days and 2 months after the diagnosis of PC. In one of these dogs, radiography of the abdomen was also performed. 

## 4. Discussion

In our study, 14 cases of PC were ultrasonographically diagnosed in 10 cats and 4 dogs over a 5-year period of study; this finding corresponds to a low prevalence of this specific form of cystitis and may confirm that PC is a rare condition in cats and also in dogs. 

In veterinary medicine, the diagnosis of PC is currently based on the ultrasonographic visualization of intraluminal pseudomembranes, which are easy to detect by ultrasound, because they tend to coat the mucosal surface. These pseudomembranes tend to adhere to the bladder mucosa and create bladder compartments, causing different ultrasound patterns, as observed in our study. In cats, all four types of adhesion were described, and an association with acoustic shadowing was observed in 60% of patients. On the contrary, the “complete adhesion” pattern, and the associated acoustic shadowing were not reported in dogs. 

This discrepancy in the ultrasonographic appearance between cats and dogs may suggest the presence of a more severe presentation of PC in the feline group. To support this hypothesis, it is important to notice that five out of ten (50%) of the cats were hospitalized due to UO and three of the remaining 50% experienced at least one previous episode of UO; on the contrary, UO was reported in none of the dogs. Indeed, UO may cause trauma of the bladder wall and enhance bladder inflammation, as a result of the dramatic increase in the intra-bladder pressure [21,22]. This hypothesis may be reinforced by the findings of the experimental study of Lavelle JP and colleagues, in which the hydrodistension of the urinary bladder of cats with feline idiopathic cystitis (FIC) caused several areas of mucosal hemorrhage [23]. Moreover, in humans, the over-distension of the bladder has been reported as a cause of bladder’s wall necrosis, due to ischemia of the wall [10,11,15,16].

Furthermore, hyperechoic pericystic fat and pericystic effusion were observed, respectively, in 80% and 40% of cats, but only in one dog. Considering that both these ultrasound abnormalities have been associated with bladder inflammation [2], this finding may suggest a greater degree of bladder inflammation in cats. Finally, this theory may also be confirmed by the higher frequency of clinical signs recorded in cats, which often presented both non-specific and specific urological signs. However, due to the small sample size of the dogs’ group, further studies are needed to valid this hypothesis.

A second hypothesis is that PC represents an evolution of a chronic form of cystitis. Indeed, in all the cats of the study, the presence of intraluminal pseudomembranes was always associated with a diffuse bladder wall thickening and intraluminal sediment, which could be an expression of chronic inflammation of the bladder [4,24]. This hypothesis may be also confirmed by the high prevalence (30%) of vesicourachal diverticulum in the feline population of our study, considering that urachal anomalies have been associated with inflammatory changes of the bladder mucosa [25]. Moreover, six out of ten cats presented pseudomembranes with acoustic shadowing, which could be related to mineralization or fibrosis [26]; also, this finding could be considered an expression of chronicity. Finally, in two cats, an abdominal ultrasound was also performed within a period between 1 month and 20 days before the diagnosis of PC, in which ultrasound images showed findings suggestive of chronic cystitis, without the presence of pseudomembranes. 

As the only cat with histological exam showed a diagnosis of ulcerative cystitis, we cannot exclude a possible role of FIC in the pathogenesis of feline PC, considering that FIC is generally seen in younger and male cats, as the cats included in our study [27,28]. Regarding the dogs’ group, it is difficult to speculate on the pathogenesis due to the small number of subjects included. Interestingly, one dog developed PC after 10 days of hospitalization. This finding may support the hypothesis that PC represents the result of a chronic process.

At urinalysis evaluation, hematuria was present in all cats and dogs, as already described in the previous case reports [1,3]. Crystalluria, leukocyturia and bacteriuria were not observed in all cats, and interestingly, bacteriuria was observed in only two out of eight cats. Although Vila et al. found bacteria in the urine of the patients of their study [3], the low prevalence of bacteriuria in our feline population may suggest a cause rather than a bacterial infection. Indeed, this finding is consistent with the fact that most of the feline low urinary tract disease in young and healthy cats is idiopathic and not associated with urinary tract infection [27].

The medical therapy was the most used in our study and in all patients was successful in resolving the clinical signs, where only one cat had a new episode of UO. Moreover, in one cat and two dogs, ultrasound images were available during the follow up. At the ultrasound examination, they presented an improvement of the bladder ultrasound aspect, with the disappearance of pseudomembranes and persistence only of ultrasound signs of chronic cystitis and some intraluminal hyperechoic material free to move. Interestingly, in one of these dogs, radiography of the abdomen was also performed, and the X-ray image showed the presence of a small amount of intraluminal mineralized vesical material, also visible in the ultrasound examination. These findings are in agreement with the case report of Vila et al. and Nevins et al, where all the patients with PC were successfully treated with medical treatment [2,3], and this suggests that the medical management may be a less invasive, conservative and effective approach for PC, although the recovery of the pathology is slow, and the recurrence of cystitis may develop in some patients.

In only one cat in our study, a cystotomy was performed to allow the removal of the bladder content. In that specific case, the cat was not able to empty the bladder independently, due to the severe compartmentalization of the bladder lumen by the pseudomembranes, and it underwent a cystotomy after 3 days of hospitalization. The surgical therapy was also used by Le Boedec et al. and it was successful [1]; however, this kind of approach could have some complications, such as the development of uroabdomen, bladder hematoma, urinary tract infection or surgical site infection [29,30,31].

A limitation of this study is its retrospective nature. Indeed, it was not possible to repeat ultrasound examinations during the follow up in all patients to verify the modalities and times of evolution of the disease.

## 5. Conclusions

In the present study, PC was confirmed to be a rare disorder in cats and also in dogs, with some ultrasonographic differences between the two species, which suggested a greater severity of the pathology in cats. Although the exact pathogenesis remains unknown, in cats, UO and relapsing chronic cystitis may have a potential role in the development of PC. Finally, in our cohort of patients, PC had a benign prognosis and based on our findings, the medical approach can be a non-invasive and effective approach for PC. In conclusion, in patients affected by PC, ultrasound is fundamental for the diagnosis and may be a very useful tool to assess the evolution of PC over time, and to monitor associated complications, such as chronic cystitis and UO.

## Figures and Tables

**Table 1:** Types of adhesion of the pseudomembranes to the bladder wall and ultrasound appearance.

Pseudomembranes Type of Adhesion		Ultrasound Appearence
Type 1	Complete adhesion	Almost completely adhered to the bladder wall, creating a coating of the mucosa, without compartmentalization of the bladder lumen
Type 2	Partial adhesion with compartmentalization	Multiple intraluminal hyperechogenic septa, creating a compartmentalization of the bladder lumen
Type 3	Partial adhesion without compartmentalization	Multiple intraluminal hyperechogenic strips, floating into the lumen, without creating a compartmentalization
Type 4	Mixed partial adhesion	Simultaneous presence of pseudomembranes described for the Type 2 and 3

**Table 2:** Abnormal ultrasound findings for the urinary bladder and pericystic peritoneal space in all patients.

Bladder Findings	Cats (*n* = 10)	Dogs (*n* = 4)
Intraluminal pseudomembranes		
• Adhesion		
○ Type 1	3 (30%)	0
○ Type 2	3 (30%)	1 (25%)
○ Type 3	2 (20%)	1 (25%)
○ Type 4	2 (20%)	2 (50%)
• Acoustic shadowing	6 (60%)	0
Sediment	10 (100%)	4 (100%)
Bladder thickening (diffuse)	10 (100%)	4 (100%)
Vesicourachal diverticulum	3 (30%)	0
Pericystic effusion	4 (40%)	1 (25%)
Hyperechoic pericystic fat	8 (80%)	1 (25%)

## Data Availability

Data are available in the clinical database of the Veterinary Teaching Hospital “Mario Modenato” (Department of Veterinary Sciences—University of Pisa).
